# Social Burden and Healthcare Costs of Colorectal Cancer

**DOI:** 10.3390/cancers17223678

**Published:** 2025-11-17

**Authors:** Izabela Gąska, Aleksandra Czerw, Monika Pajewska, Olga Partyka, Andrzej Deptała, Anna Badowska-Kozakiewicz, Natalia Czerw, Dominika Mękal, Katarzyna Sygit, Klaudia Malikowska, Jarosław Drobnik, Piotr Pobrotyn, Dorota Waśko-Czopnik, Tomasz Sowiński, Ewa Bandurska, Weronika Ciećko, Elżbieta Grochans, Anna Maria Cybulska, Daria Schneider-Matyka, Kamila Rachubińska, Petre Iltchev, Tomasz Czapla, Remigiusz Kozlowski

**Affiliations:** 1 Medical Institute, Jan Grodek State University in Sanok, 38-500 Sanok, Poland; 2 Department of Health Economics and Insurance, Center for the Humanities and Social Sciences of Medicine, Medical University of Warsaw, 00-581 Warsaw, Poland; 3 Department of Economic and System Analyses, National Institute of Public Health NIH-National Research Institute, 00-791 Warsaw, Poland; 4 Department of Oncology Propaedeutics, Medical University of Warsaw, 01-445 Warsaw, Poland; 5 Students’ Scientific Organization of Cancer Cell Biology, Department of Oncology Propaedeutics, Medical University of Warsaw, 01-445 Warsaw, Poland; 6 Faculty of Medicine and Health Sciences, University of Kalisz, 62-800 Kalisz, Poland; 7 Department of Family Medicine, Faculty of Medicine, Wroclaw Medical University, 51-141 Wroclaw, Poland; 8 Pulsantis Specialist and Rehabilitation Clinic Ltd., 53-238 Wroclaw, Poland; 9 Department of Gastroenterology, Hepatology with Inflammatory Bowel Disease Subunit, Provincial Specialist Hospital J. Gromkowskiego, 51-149 Wroclaw, Poland; 10 Department of Non-Surgical Clinical Sciences, Faculty of Medicine, Wrocław University of Science and Technology, 50-370 Wroclaw, Poland; 11 Endocare Medical Center, Simple Joint-Stock Company (S.J.S.C.), 50-558 Wroclaw, Poland; 12 Center for Competence Development, Integrated Care and e-Health, Medical University of Gdansk, 80-204 Gdansk, Poland; 13 Department of Nursing, Faculty of Health Sciences, Pomeranian Medical University in Szczecin, 71-210 Szczecin, Poland; 14 Department of Management and Logistics in Healthcare, Medical University of Lodz, 90-136 Lodz, Poland; petre.iltchev@umed.lodz.pl (P.I.); remigiusz.kozlowski@umed.lodz.pl (R.K.); 15 Department of Management, Faculty of Management, University of Lodz, 90-237 Lodz, Poland; tomasz.czapla@uni.lodz.pl

**Keywords:** colorectal cancer, CRC, costs, social burden, direct costs, indirect costs, cost-effectiveness

## Abstract

Background: Colorectal cancer is the third most common malignant cancer, and according to the predictions, the estimated number of new cases will grow in coming years. Therefore, an increase in the costs of the disease will increase as well. Therefore, there is a need for continuous research on the costs and the economic burden of colorectal cancer and for reviewing the research results systematically. The current paper presents a literature review regarding the state of knowledge about the costs of treatment and the economic burden of colorectal cancer. Methods: A total of 20 papers from MEDLINE database were included in the final analysis. The review is focused on the estimates of direct costs, i.e., treatment of colorectal cancer, screening after treatment, and indirect costs. Results: the vast majority of studies were focused on direct costs only, which clearly shows the literature gap. Metastatic colorectal cancer was the most frequent category for various treatment cost evaluation. The costs associated with the use of bevacizumab in various combinations were calculated most frequently. Conclusions: Further summarizing review and developing a methodology for standardized comparisons is necessary, specifically addressing indirect costs.

## Simple Summary

Colorectal cancer presents a growing global challenge, not only in terms of public health but also economic burden. This article offers a comprehensive review of recent literature focused on the financial impact of CRC, analyzing both direct medical expenses and the less frequently examined indirect costs. Most studies concentrate on treatment costs, particularly for metastatic cases highlighting frequent use of targeted therapies like bevacizumab. Significant disparities in cost effectiveness emerge across countries, influenced by healthcare systems, drug pricing, and cancer staging. Despite the long-term and often debilitating nature of CRC, only a minority of studies account for indirect costs such as lost productivity or social burden, revealing a critical gap in economic analysis. Similarly, post treatment surveillance strategies receive limited attention. With projections indicating a sharp rise in CRC incidence especially in low and middle income regions, the need for standardized, crossnational methods of cost evaluation is increasingly urgent.

## 1. Introduction

Colorectal cancer is the third most common malignant cancer worldwide, following lung cancer and breast cancer. In terms of mortality, colorectal cancer ranks second, following lung cancer. According to data from the World Health Organization, there were 1,926,425 new cases worldwide in 2022. The age-standardized incidence rate was 10.7 per 100,000 people [1]. Denmark recorded the highest incidence rate, reaching 48.1 per 100,000. In the same year, 538,167 deaths were reported globally, with an age-standardized mortality rate of 4.7 per 100,000. The five-year survival rate exceeds 50% in high-income countries, varying between 59.0% in the United Kingdom and 70.8% in Australia. Projections from the International Agency for Research on Cancer [2] suggest that the number of new cases will rise significantly in the coming years, particularly across Africa, Latin America, Oceania, and Asia (see Table 1). The expected growth is a reason for continuous research on the costs of the treatment and the economic burden of colorectal cancer and for reviewing the research results systematically.

Main risk factors for colorectal cancer include getting older, specifically over 50 years old, family history of colorectal cancer or Lynch syndrome, familial adenomatous polyposis, having colorectal cancer before or certain types of polyps in personal history, a diet rich in processed meats and low in fruits and vegetables, sedentary behavior, obesity, smoking, and excessive alcohol consumption [3].

Diagnostics and screening tests include fecal occult blood tests, sigmoidoscopy, digital rectal exam, and colonoscopy [4]. The treatment methods of colorectal cancer include surgery, radiotherapy, chemotherapy, targeted therapy, and immunotherapy.

Colorectal cancer is one of the chronic diseases and it requires long-term treatment, which is expensive and usually limits the social and professional life of patients. The medical costs associated with colorectal cancer treatment were previously [5] estimated for four consecutive stages. The initial mean 3-year costs, including the costs of diagnosis, surgery, radiotherapy, and chemotherapy were estimated to be equal to EUR 7754 for stage I, EUR 8494 for stage II, and to EUR 8260 for stage III, so the costs for stage II and III were higher than the costs for stage I. Follow-up costs, including hospitalization, outpatient consultations, diagnostic tests, chemotherapy, or pharmacological treatments, were estimated to be equal to EUR 2688 for stage I, EUR 3159 for stage II, and to EUR 3428 for stage III, so the follow-up costs were higher for more advanced stages of colorectal cancer. The mean 3-year costs of stage IV colorectal cancer, including diagnosis, in-hospitalizations, radiotherapy, chemotherapy, and follow-up consultations were estimated to be equal to EUR 19,423.

Two main categories of costs should be taken into account: direct and indirect costs. The direct costs primarily encompass healthcare system expenditures related to medical services, as well as patients’ own expenses associated with treatment, including out-of-pocket payments for medications and medical supplies. Indirect costs refer to productivity losses resulting from sickness absence or reduced work efficiency (presenteeism) [6]. As projections from the Global Cancer Observatory suggest an increasing incidence of colorectal cancer, the growing number of patients is expected to generate higher treatment-related expenditures and greater productivity losses due to illness-related absences. Attention should also be paid to the existence of health debt after the COVID-19 pandemic caused by, among others, limited access to screening programs during this period, which will generate additional financial burden on systems whose operation varies significantly between countries [7,8].

The objective of this article is to present the latest available knowledge on the direct and indirect costs of colorectal cancer as a growing problem for healthcare financing.

## 2. Materials and Methods

A literature review was conducted to summarize the current state of knowledge regarding the treatment costs and economic burden of colorectal cancer using the MEDLINE database. The following search strategy was applied: (colorectal cancer) AND (cost OR costs OR economic analysis OR economic evaluation OR economic loss OR expenditure OR spending OR expense OR burden OR productivity OR cost analysis). The search was restricted to articles published in 2024 and 2025. Additional filters were used to include only studies involving human subjects and addressing clinical questions related to costs and economic aspects. By applying the criteria we aimed at finding the latest research results that relate directly to the costs associated with colorectal cancer. The criteria for inclusion of publications according to the PICOS scheme are presented in Table 2.

**Figure 1:**
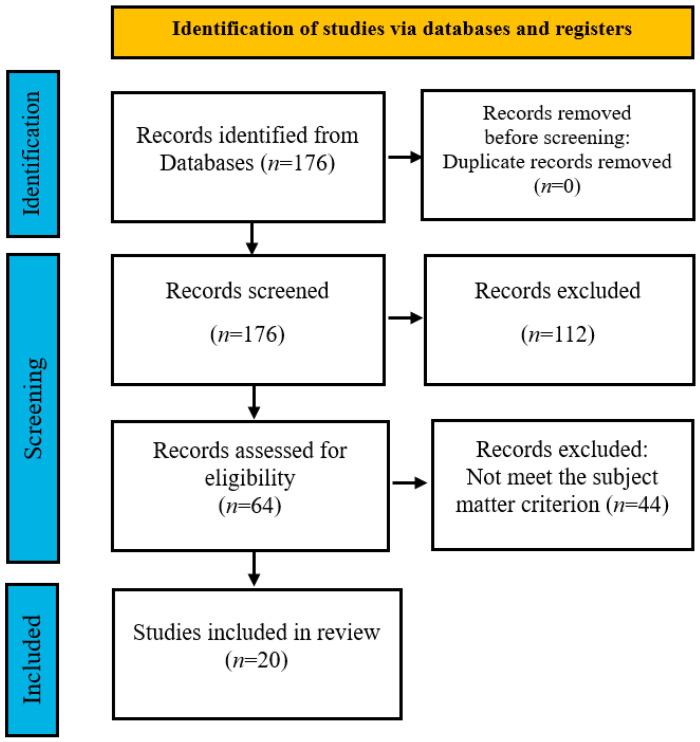
Simplified PRISMA scheme for inclusion of publications for further review.

The search performed within the records of MEDLINE database led to 176 papers. After title and abstract verification for being relevant to the subject the number of articles was limited to 64. Next, full text verification was performed and it ended up with 20 papers included in the final analysis. PRISMA schema is depicted in Figure 1.

In the subsequent sections, cost estimates are presented in the original currencies reported by the authors of the referenced studies. For studies that used currencies other than the US dollar, values were converted to USD using the annual average operational exchange rates provided by the United Nations Treasury [9].

## 3. Results

Table 3 presents a summary of the publications included in the review with a description of the most important features.

The review of the results acquired in the studies included is divided into sections for direct costs, i.e., treatment of colorectal cancer, screening after treatment, and indirect costs.

### 3.1. Direct Costs

#### 3.1.1. Treatment of Colorectal Cancer

Eleven studies evaluated cost-effectiveness of specific drugs. The first five focused on treatment involving the use of bevacizumab. The first cost-effectiveness study [10] demonstrated S-1 and oxaliplatin plus bevacizumab to be a cost-effective alternative to oxaliplatin plus cetuximab as a first-line treatment for patients with Kirsten rat sarcoma virus wild-type metastatic colorectal cancer (mCRC) in Japan. The use of oxaliplatin with bevacizumab was associated with an incremental cost equal to 6528 USD, an incremental effectiveness equal to 0.79 QALY, and an ICER equal to 8215 USD per QALY.

Bevacizumab with trifluridine/tipiracil, on the other hand, was not more cost effective than trifluridine/tipiracil monotherapy as a third-line treatment for patients with colorectal cancer in China [11]. The cost of treatment with trifluridine/tipiracil plus bevacizumab was equal to ¥838,492.74, which can be converted to 116,522.73 USD, and higher than monotherapy with trifluridine/tipiracil, though it had greater health benefits (2.45 QALYs vs. 1.54 QALYs). The ICER was equal to ¥527,577.36/QALY, which can be converted to USD 73,315.79/QALY.

Continuation of bevacizumab plus chemotherapy as a second-line treatment was evaluated in China [12]. The continuation was associated with an increase equal to 0.12 QALYs and an incremental cost of 22,761.62 USD compared with chemotherapy alone, resulting in an ICER of 188,904.09 USD per QALY, and it was considered not cost effective for patients after first progression of metastatic colorectal cancer. The estimates were based on the data from 820 patients.

Bevacizumab biosimilars, MVASI and Zirabev, were associated with real-world cost savings in use as the first-line treatment of metastatic colorectal cancer while providing similar survival benefit as the originator [13]. The biosimilars were associated with an incremental cost of −6379 USD (95%CI: −9417, −3537) (negative values mean cost saving) and incremental effect of 0.0 (95% CI: −0.02, 0.02) LY and −0.01 (95% CI: −0.03, 0) quality-adjusted LYs gained. Incremental net monetary benefit was 6331 USD (95% CI: 6245, 6417). The estimates were based on the data from 3242 patients.

Cost-effectiveness analysis of treating metastatic colorectal cancer in first-line therapy with XELOX versus XELOX plus bevacizumab was evaluated in Brazil [14]. The difference in years of life gained was equal to just 2.25 months, with an extra cost of 47,833.57 BRL, which can be converted into 14,951.88 USD, resulting in an incremental cost-effectiveness of 21,231.43 BRL, which can be converted into 6636.55 USD per month of life gained. The estimates were based on the data from three studies.

The other studies were focused on other types of treatment. The cost effectiveness of treating patients with refractory metastatic colorectal cancer with fruquintinib was evaluated in China [15]. The total cost for the fruquintinib was equal to 11,089.05 USD and compared with a placebo group, i.e., 5374 USD. The overall QALYs were higher for fruquintinib group (0.61 QALYs vs. 0.43 QALYs for placebo). The ICER was equal to 31,747.67 USD per QALY. The estimates were based on the data from 691 patients.

The cost-effectiveness of FOLFOXIRI/FOLFOXIRI compared with mFOLFOX6/FOLFIRI in first-line and second-line chemotherapy for metastatic colorectal cancer from the United States and China was evaluated in another study [16]. Treating patients with FOLFOXIRI/FOLFOXIRI was associated with 0.08 QALYs in the USA and with 0.04 QALYs in China when compared with the mFOLFOX6/FOLFIRI. The ICERs for FOLFOXIRI/FOLFOXIRI were 5127.70 USD per QALY in the United States and 30,478.33 USD per QALY in China. Treatment with FOLFOXIRI/FOLFOXIRI as first-line and second-line chemotherapy was more efficient financially. The estimates were based on the data from 691 patients recruited from 58 Italian oncology units.

5-fluorouracil is used in first-line chemotherapy for metastatic colorectal cancer. The cost-effectiveness of pharmacokinetics-guided vs. body surface area dosing for patients with metastatic colorectal cancer was evaluated in Australia [17]. Pharmacokinetics-guided was found to provide better health outcomes and lower costs. Body surface area dosing provided 1.291 QALYs at a cost of 36,379 USD; pharmacokinetics-guided provided 1.751 QALYs at a cost of 32,564 USD. The estimates were based on the data from 157 patients.

TAS-102 and regorafenib were not found to be cost effective compared to supportive care alone [18]. Regorafenib was associated with ICER of 395,223 USD per quality-adjusted life year and 399,740 USD per QALY. TAS-102 was associated with 399,740 USD per QALY.

The next two studies focused on cost-effectiveness analysis for treatments developed for patients with metastatic colorectal cancer with mutated KRAS G12C. Cost effectiveness of treatment sotorasib with panitumumab compared to standard care in cases of treatment of refractory colorectal cancer with mutated KRAS G12C was evaluated in another study [19]. However, no economic advantage compared to standard care was found. The estimated benefit was 0.876 QALYs vs. 0.857 QALYs for standard care. The ICER was equal to 3,551,555,554 USD/QALY.

The cost-effectiveness of treating colorectal cancer patients with mutated KRAS G12C with adagrasib and cetuximab in comparison to adagrasib alone was evaluated in another study [20]. Treatment with adagrasib and cetuximab was not found to be cost effective for advanced or metastatic colorectal patients with mutated KRAS G12C. The cost of treatment with adagrasib and cetuximab was equal to 290,645.434 USD with 1.094 QALYs and higher than that of treatment with adagrasib alone (188,837.346 USD) with 1.359 QALYs. The ICER was calculated at −384,674.32 USD/QALY.

**Figure 2:**
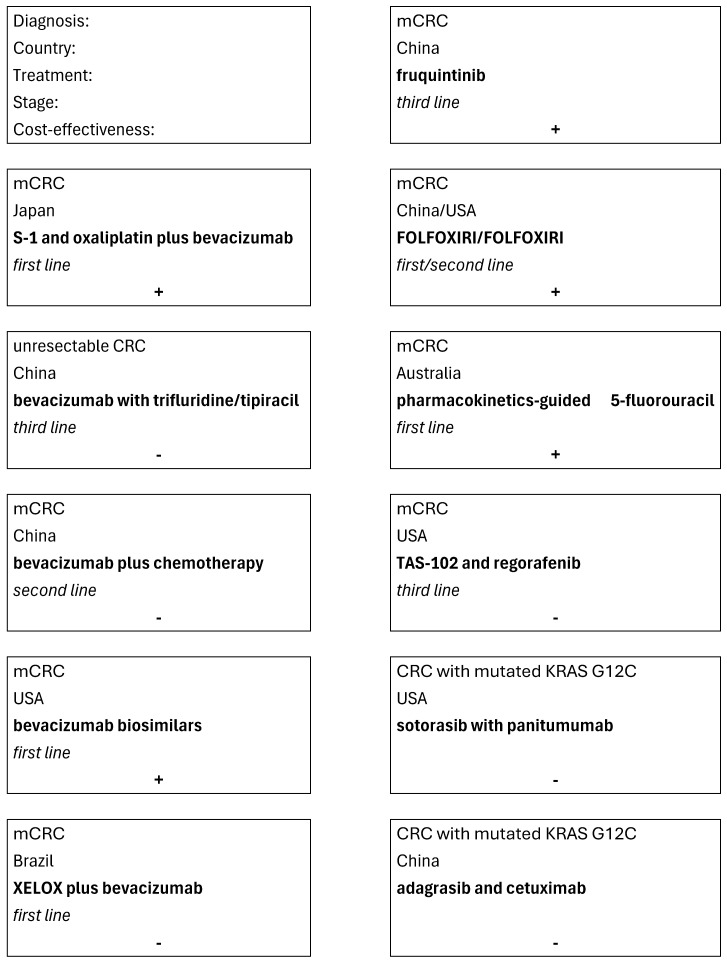
Summary of the studies examining the cost-effectiveness of drugs. Note: Text first in text box depicts the order of information.

On the basis of studies presented above, one can conclude that the treatments based on S-1 and oxaliplatin plus bevacizumab, bevacizumab biosimilars, fruquintinib, FOLFOXIRI/FOLFOXIRI, and pharmacokinetics-guided 5-fluorouracil were cost effective. The treatments based on bevacizumab with trifluridine/tipiracil, bevacizumab plus chemotherapy, XELOX plus bevacizumab, TAS-102 and regorafenib, sotorasib with panitumumab, and on adagrasib and cetuximab were not found to be cost effective. The results of the studies examining the cost effectiveness of drugs are summarized on Figure 2.

The papers discussed below were focused on estimating the costs of treatment. Another study estimated the cost values with the effectiveness in each stage of colorectal cancer in Romania [21]. The mean ICER values were equal to 4231.37 EUR per year of survival in stage I, 4556.76 EUR in stage II, 2196.78 EUR in stage III, and 175,703.29 EUR in stage IV, which can be converted to 4574.25 USD per year of survival in stage I, 4926.01 USD in stage II, 2374.79 USD in stage III, and 189,940.95 USD in stage IV. The estimates were based on the data from 423 patients.

Costs were found to rise with advanced cancer stages [22]. Patients’ average out-of-pocket expenses in Australia are equal roughly to $A441 per year, which can be converted to 290.70 USD. On average, males spend 13.5% higher annually. Patients aged 50–70 are involved in spending 7.1% more than patients aged 50 or below. Patients older than 70 spend less than patients aged 50 or below by 8.8%, which can be associated with applying less intensive treatment for older patients. The estimates were based on the data from 29,892 patients.

The costs of colorectal cancer in the group of patients with Type 2 Diabetes Mellitus were found to be substantially higher [23]. Colorectal cancer in this group was associated with higher direct medical costs (¥57,059.65 equal to 7882.57 USD vs. ¥48,933.93 equal to 6760.03 USD, *p* < 0.05), direct nonmedical costs (¥9292.45 equal to 1283.72 USD vs. ¥7969.35 equal to 1100.93 USD), and total disease costs (¥70,253.93 equal to 9705.31 USD vs. ¥59,776.36 equal to 8257.87 USD, *p* < 0.05) when compared to nondiabetic patients. The estimates were based on the data from 818 patients.

The costs associated with young-onset colorectal cancer were substantially higher than if the onset was average [24]. The overall average annualized costs were equal to the initial 50,216 USD (95% CI: 47,853–52,851), continuing 8361 USD (95% CI: 7155–9573), and end-of-life cancer phase 86,125 USD (95% CI: 80,319–92,751) compared to the initial 37,842 USD (95% CI: 37,153–38,560), continuing 5014 USD (95% CI: 4727–5327) and end-of-life cancer phase 61,512 USD (95% CI: 59,197–63,530) when the onset was average. The estimates were based on the data from 13,677 patients.

The annual direct medical cost for colorectal cancer in Antigua and Barbuda was estimated to equal to USD 1.14 million, with treatment (USD 613,650.01) and post-treatment side-effects care (USD 402,234.50) as the main expenditures [25]. The total estimated direct medical unit costs amounted to USD 139,295.58. The primary cost components included surgical procedures (USD 43,467.10), treatment-related complications (USD 28,469.21), and immunotherapy (USD 19,200.00). The estimates were based on the data from 158 patients.

The estimated overall economic burden of colorectal cancer in China was equal to ¥170.5 billion, which can be converted to 23.69 billion USD and is 0.189% of the local GDP [26]. The direct costs were equal to ¥106.4 billion, which can be converted to 14.79 billion USD and is 62.4% of the total economic burden.

In Belgium colorectal cancer was associated with average incremental cost of EUR 4030 (95% CI: 3925–4135) [27], which can be converted into 4356.56 USD. Total cost was estimated to be equal to 282,290,062 with total years of life lived with disability equal to 7.233.

In Japan the cost of molecular targeted drugs containing regimens for patients with metastatic colorectal cancer fall in the range from 85,406 to 843,602 JPY per month, which can be converted to the range from 563,07 USD to 5561.78 USD, and the cost of cytotoxic drug regimens only fall in the range from 17,672 to 51,004 JPY per month [28], which can be converted to the range from 116.51 USD to 336.23 USD. About 16% of metastatic colorectal cancer patients were treated in the first line with regimens costing >500,000JPY per month (equal to >3296.45 USD per month). The molecular targeted drugs were the main expenditure. The estimates were based on the data from 1880 patients.

Advanced stage, young-onset, and type 2 Diabetes Mellitus were found to increase the costs of colorectal cancer in the studies discussed above.

The papers in 2024 and 2025 did not evaluate the cost-effectiveness of epidermal growth factor receptor (EGFR) inhibitors. The clinical effectiveness of this treatment for metastatic colorectal cancer with RAS and BRAF wild-type cancer is limited due to primary and acquired resistance. However, extended genetic profiling may help to exclude tumors which are unlikely to benefit from EGFR therapy [29,30]. Also, cost-effectiveness analysis of first-line FOLFIRI combined with cetuximab or bevacizumab from 2020 [31] revealed cost-effective probability of cetuximab under the willingness-to-pay threshold of $24,081 in China.

#### 3.1.2. Screening After Treatment

The strategies of surveillance during the first five years after curative resection of colorectal cancer were evaluated in Japan [29]. The most effective strategy was found to be providing clinical evaluation and laboratory investigations 6, 12, 18, 24, 36, 48, and 60 months after the treatment and computed tomography 12, 24, 36, 48, and 60 months after the treatment. Colonoscopy was to be provided 12 and 36 months after the treatment. This strategy was associated with ICER of 26,555 USD/quality-adjusted life year. The estimates were based on the data from 3701 patients.

### 3.2. Indirect and Social Costs

The social costs of CRC represent a major component of the disease’s total economic burden, primarily through lost productivity, disability, and premature mortality. These indirect costs are particularly significant given the long-term nature of CRC treatment and its impact on patients’ ability to work and engage in everyday life.

According to Wang et al., the estimated indirect costs of CRC in China alone amounted to ¥64.1 billion, which can be converted to 8.91 billion USD, with 63.7% attributed to productivity losses due to premature death [26]. Moreover, patients with comorbidities such as type 2 diabetes mellitus experience substantially higher social costs, with indirect expenses reaching ¥300.13 compared to ¥241.11 in non-diabetic patients [23], which can be converted to 41.46 USD and 33.31 USD, respectively. In Australia out-of-pocket expenses for CRC patients averaged $A441 annually, with working-age adults (50–70 years) incurring 7.1% more costs, reflecting the impact on individuals in their most productive years [22]. It can be converted to 290.70 USD.

Despite their significance, these indirect and social costs remain underrepresented in the literature; only a few of the reviewed studies explicitly quantified them, underscoring the need for broader inclusion of social costs in CRC economic evaluations.

## 4. Limitations

The scope of the review is limited to the papers included in MEDLINE database only, which may underrepresent some relevant evidence and cost components reported elsewhere. Since the estimates of direct costs in different studies use different units depending on the currency specific to a region, each paper is focused on the comparisons, which are difficult, as the relative value of each currency when exchanged to another currency is not constant but changes over time. The currencies in the current review were converted to USD. However, this conversion is approximate only. For indirect costs, estimates are inherently country-specific and depend on valuation methods and labor market assumptions, which limits generalizability across settings. Also, the accessibility of papers focused on indirect costs is limited, which is a key limitation as well. The limitations mentioned above pose a challenge in evaluating costs of various types of cancer, including colorectal cancer.

## 5. Conclusions

The vast majority of papers focus on direct costs of pharmacological treatment. Five studies out of twenty were focused on comparisons within the same drug class. However, comparing the cost-effectiveness of different treatment approaches is challenging due to the complexity arising from the use of diverse cost assessment units across studies. This difficulty is further compounded by variations between countries and by the fact that cost effectiveness is strongly influenced by the specific diagnostic category and the stage of the disease. Just one study used a placebo group. The results of five studies led to favorable conclusions. The treatments based on S-1 and oxaliplatin plus bevacizumab, bevacizumab biosimilars, fruquintinib, FOLFOXIRI/FOLFOXIRI, and pharmacokinetics-guided 5-fluorouracil were found to be cost effective. Two studies focused on direct costs depending on a clinical condition, i.e., the early or regular onset of the disease or comorbid diabetes. Advanced stage, young-onset, and type 2 Diabetes Mellitus were found to be associated with higher costs.

Only one out of twenty studies was focused on screening after treatment, and just two provided estimates of indirect costs. The indirect costs are clearly overlooked, so there is a literature gap for evaluating indirect costs of colorectal cancer. The studies focused on direct costs of treatment took specific types of treatment into consideration. The use of bevacizumab and the diagnosis of metastatic colorectal cancer were most frequently investigated.

Five studies provided estimates of direct costs of treatment from the perspective of a country, each using a different currency. The comparison of indirect costs is also difficult because of the various units of currency used for the estimates.

Unfortunately, the cost-effectiveness studies do not refer directly to guidelines for treatment used in clinical practice [32]. The use of 5-fluorouracil + folinic acid or CAPOX in the course of adjuvant therapy was not evaluated. The cost effectiveness of immunotherapy in use in cases of high microsatellite instability was evaluated in single study [24]. Other literature gaps involve the treatment of colorectal cancer with HER-2 amplification and a KRAS G12C mutation with panKRAS inhibitors.

The estimated number of new colorectal cancer cases is expected to rise in the coming years, inevitably leading to an increase in all categories of associated costs. Therefore, the development of a standardized methodology for comparison, synthesis, and evaluation of cost-related data is essential.

## Figures and Tables

**Table 1:** Estimated number of new cases of colorectal cancer from 2022 to 2045, aged 0–85+.

Year	Africa		Latin America and the Caribbean		Northern America		Europe		Oceania		Asia	
	n	Risk (%)	n	Risk (%)	n	Risk (%)	n	Risk (%)	n	Risk (%)	n	Risk (%)
2022	70,428	0	145,120	0	183,973	0	538,262	0	22,243	0	966,399	0
2025	77,410	9.9	152,882	5.3	194,879	5.9	552,399	2.6	24,383	9.6	1,046,155	8.3
2030	91,835	30.4	177,077	22.0	211,254	14.8	592,761	10.1	27,683	24.5	1,209,940	25.2
2035	108,656	54.3	203,323	40.1	226,382	23.1	629,311	16.9	31,032	39.5	1,383,209	43.1
2040	127,972	81.7	230,655	58.9	239,441	30.2	659,365	22.5	34,229	53.9	1,553,816	60.8
2045	149,855	112.8	258,059	77.8	250,208	36.0	681,867	26.7	37,299	67.7	1,714,698	77.4
2050	174,396	147.6	284,828	96.3	259,177	40.9	696,727	29.4	40,280	81.1	1,866,959	93.2

Source. Global Cancer Observatory. n—number of new cases; Risk (%)—risk percentage, i.e., number of new cases in the year of analysis as a percentage of year 2022 case base.

**Table 2:** Criteria for inclusion of publications according to the PICO scheme.

Population (P)	Patients Diagnosed with Colorectal Cancer
Intervention (I)	Costs, Economics
Comparator (C)	Any or none
Outcomes (O)	Direct costs of colorectal cancer treatment, indirect costs of colorectal cancer, economic burden
Studies (S)	Case studies, prospective studies, retrospective studies, systematic review, RCT
Limitations	Publications in English assessing the impact of colorectal cancer on the quality of life, publication period 01.01.2023–30.05.2025
Exclusion	Non-English publications, studies not directly linked to colorectal cancer

**Table 3:** Characteristics of publications included in the review.

Author/Year	Country	Unit of Measure	Currency	Methodology	Type of Costs	Group of Patients
Morimoto, T. et al., 2025 [10]	Japan	QALY, ICER	USD	Cost-effectiveness analysis	Direct costs	Patients with Kirsten rat sarcoma virus wild-type metastatic colorectal cancer
Huang, L. et al., 2024 [11]	China	QALY, ICER	CNY	Cost-effectiveness analysis	Direct costs	Patients with colorectal cancer
Li, Y. et al., 2024 [12]	China	QALY, ICER	USD	Cost-effectiveness analysis	Direct costs	Patients with metastatic colorectal cancer
Lu, B. et al., 2024 [13]	United States	QALY, ICER	USD	Cost-effectiveness analysis	Direct costs	Patients with metastatic colorectal cancer
Ungari, A. et al., 2017 [14]	Brazil	QALY, ICER	BRL	Cost evaluation	Direct costs	Patients with metastatic colorectal cancer
Huang, Z. et al., 2024 [15]	China	QALY, ICER	USD	Cost-effectiveness analysis	Direct costs	Patients with refractory metastatic colorectal cancer
Li, X. et al., 2025 [16]	China, United States	QALY, ICER	USD	Cost-effectiveness analysis	Direct costs	Patients with metastatic colorectal cancer
Erku, D. et al., 2025 [17]	Australia	QALY, ICER	USD	Cost evaluation	Direct costs	Patients with metastatic colorectal cancer
Cho, S. et al., 2018 [18]	United States	QALY, ICER	USD	Cost-effectiveness analysis	Direct costs	Patients with metastatic colorectal cancer
Liu, T. et al., 2025 [19]	United States	QALY, ICER	USD	Cost-effectiveness analysis	Direct costs	Patients with metastatic colorectal cancer with mutated KRAS G12C
Yao, R. et al., 2025 [20]	United States	QALY, ICER	USD	Cost-effectiveness analysis	Direct costs	Patients with metastatic colorectal cancer with mutated KRAS G12C
Gana, A. et al., 2024 [21]	Romania	ICER	EUR	Cost evaluation	Direct costs	Patients with colorectal cancer
Yang, O. et al., 2024 [22]	Australia	-	AUD	Cost evaluation	Direct costs	Patients with colorectal cancer
Zhu, Z. et al., 2025 [23]	China	QALY	CNY	Cost evaluation	Direct and indirect costs	Patients with colorectal cancer and Type 2 Diabetes Mellitus
Garg, R. et al., 2024 [24]	United States	-	USD	Cost evaluation	Direct costs	Patients with young onset colorectal cancer
Bovell, A. et al., 2025 [25]	Antigua and Barbuda	-	USD	Cost evaluation	Direct costs	Patients with colorectal cancer
Wang, H. et al., 2024 [26]	China	-	CNY	Cost evaluation	Direct and indirect costs	Patients with colorectal cancer
Gorasso, V. et al., 2024 [27]	Belgium	-	EUR	Cost evaluation	Direct costs	Patients with colorectal cancer
Takashima, A. et al., 2024 [28]	Japan	-	JPY	Cost evaluation	Direct costs	Patients with metastatic colorectal cancer
Takayama, Y. et al., 2024 [29]	England	ICER	USD	Cost-effectiveness analysis	Direct costs	Patients after curative resection of colorectal cancer

## Data Availability

Data available from authors.
